# Wearable Hearing Device Spectral Enhancement Driven by Non-Negative Sparse Coding-Based Residual Noise Reduction

**DOI:** 10.3390/s20205751

**Published:** 2020-10-10

**Authors:** Seon Man Kim

**Affiliations:** Korea Photonics Technology Institute, Gwangju 61007, Korea; smkim@kopti.re.kr; Tel.: +82-62-605-9262; Fax: +82-62-605-9259

**Keywords:** hearing device, hearing aid, speech enhancement, Wiener filter, residual noise, non-negative sparse coding

## Abstract

This paper proposes a novel technique to improve a spectral statistical filter for speech enhancement, to be applied in wearable hearing devices such as hearing aids. The proposed method is implemented considering a 32-channel uniform polyphase discrete Fourier transform filter bank, for which the overall algorithm processing delay is 8 ms in accordance with the hearing device requirements. The proposed speech enhancement technique, which exploits the concepts of both non-negative sparse coding (NNSC) and spectral statistical filtering, provides an online unified framework to overcome the problem of residual noise in spectral statistical filters under noisy environments. First, the spectral gain attenuator of the statistical Wiener filter is obtained using the a priori signal-to-noise ratio (SNR) estimated through a decision-directed approach. Next, the spectrum estimated using the Wiener spectral gain attenuator is decomposed by applying the NNSC technique to the target speech and residual noise components. These components are used to develop an NNSC-based Wiener spectral gain attenuator to achieve enhanced speech. The performance of the proposed NNSC–Wiener filter was evaluated through a perceptual evaluation of the speech quality scores under various noise conditions with SNRs ranging from -5 to 20 dB. The results indicated that the proposed NNSC–Wiener filter can outperform the conventional Wiener filter and NNSC-based speech enhancement methods at all SNRs.

## 1. Introduction

Individuals with hearing impairment often have trouble understanding the specific meaning of speech in their everyday lives. Researchers have attempted to solve this issue by developing wearable hearing aid devices, which are commonly used to balance the dynamic range to compensate for hearing loss [1]. However, many individuals find the functioning of hearing aids to be inadequate, mostly owing to the interference of noise with the speech signal entering the ear. In particular, only 23% of hearing-impaired (HI) (All the abbreviations used in this paper are listed in the Abbreviations) individuals use hearing aid devices [2,3]. The limitations associated with noisy speech in the context of hearing aids were reported more than 35 years ago [4] and have not yet been effectively addressed.

A potential solution is to use multiple microphones, which can improve the signal-to-noise ratio (SNR); however, this improvement is limited by several factors. In real-life situations, hearing aids cannot function adequately in environments involving multiple noise sources and high reverberation [5]. Moreover, the size of modern hearing aids is continually decreasing, owing to which, only one or two microphones can be installed. Consequently, single-channel noise reduction algorithms have been developed to facilitate the complex speech perception for hearing aid users.

Speech enhancement algorithms improve the quality of the target speech signal by reducing the effects of background noise, thereby notably reducing the amount of listening effort and fatigue that the background noise requires of the listener. Typically, this enhancement is realized by retaining the characteristics of speech through a short-term spectral amplitude (STSA) analysis, implemented using statistical techniques such as Wiener filters, minimum mean square error (MMSE) estimation, and MMSE log-spectral amplitude estimation, to improve the quality of noisy speech by suppressing additive background noise [5,6]. These techniques are based on the accurate estimation of the a priori SNR by estimating the noise power spectral densities (PSDs). Such statistical techniques can reduce the noise in speech under stationary noise conditions, using techniques such as the decision-directed (DD) approach [7,8]. However, it is difficult to reliably estimate the noise PSDs when speech is corrupted by non-stationary noise such as babble noise; in such cases, the a priori SNR estimations are often inaccurate because the noise components remain in the enhanced speech spectrum even after being processed through the Wiener filter [9,10].

In recent decades, in order to find more effective solutions for speech enhancement, research focus has shifted to deep neural network (DNN) techniques from the aforementioned STSA approaches [11,12]. DNN-based speech enhancement methods can notably outperform statistical approaches, even though a more complex neural network architecture is required to achieve a higher performance in speech enhancement tasks. Nevertheless, such approaches involve prohibitively high computing costs and thus cannot be easily applied in portable hearing devices (e.g., hearing aids) that require low computing complexity approaches for real-world implementation.

An alternative approach to extract clean speech from a noisy speech signal is to use a non-negative matrix factorization (NMF)-based signal decomposition technique [13,14]. In the NMF framework, the speech and noise spectral components are approximately reconstructed using the non-negative speech and noise bases, corresponding to the speech and noise, respectively [15,16]. Nevertheless, even if a sufficiently large universally representative speech database (DB) can be collected to construct the non-negative speech base dictionary, it may still be challenging to collect all real noises to acquire the noise bases in the existing noise environments [16,17]. To address this problem, the framework must be trained online by using the noise bases pertaining to the currently observed noise, and this aspect has been actively studied [16,17]. However, the performance of online training depends significantly on the accuracy of estimation of the noise intervals from the observed noisy speech, which represents another critical challenge [8].

In this context, it is desirable to develop an NMF-based approach that employs only the speech bases instead of explicitly estimating the noise bases. The NMF approach introduced in [18,19] reconstructed the clean speech spectral components from noisy speech by using only the bases from a speech dictionary with sparse activation. This non-negative sparse coding (NNSC) strategy was noted to be efficient in the speech enhancement for a cochlear implant and could enhance the speech enhancement performance, especially in noisy environments [18]. 

However, although this method could be applied to cochlear implants, it could not necessarily improve the speech quality for other hearing device applications, as described further in Section 5. In general, speech consists of harmonic and non-harmonic components across a wide range of frequencies, and the noise spectrum is also relatively widely spread over the frequency. Thus, part of the noise spectrum can be represented by the speech dictionary (or basis), and it generates a certain residual noise in the estimated target speech. 

Considering these aspects, this paper proposes an improved NNSC-based speech enhancement algorithm that reduces the residual noise based on the principle that the residual noise components remaining after processing through the DD-based Wiener filter tend to be whitened [9,10]. Furthermore, the NNSC approaches, as a statistical approach, aim to identify the basis and activation components by minimizing the Gaussian independent identically distributed noise [18]. In this context, the NNSC can reconstruct the target speech spectra of the Wiener filter output by using a pre-trained dictionary while removing the whitened residual noise components. In other words, the proposed method applies NNSC to the spectrum enhanced through Wiener filtering, thereby reducing the residual noise and minimizing the speech distortion compared to that when using only a Wiener filter or the NNSC strategy. The objective is to enhance the speech quality rather than the speech intelligibility. In general, noise reduction strategies are highly correlated with an improved speech quality, although they may not always lead to improved intelligibility [8].

Furthermore, the objective is to implement the speech enhancement algorithm on an auditory hearing device filter bank that can satisfy unique conditions such as the signal quality, computational complexity, and signal delay. In particular, the latency in auditory processing algorithms should not be more than 10 ms, to prevent a deterioration in the subjective listening experience [20,21,22,23,24]. Moreover, the approaches should have a low computational complexity due to the limited processing capacity and battery power in real-world portable devices [22,25,26]. To this end, many researchers employ a discrete Fourier transform (DFT)-based uniform polyphase filter bank, as it can enable perfect reconstruction with low latency and can be expanded into non-uniform filter banks [20,21,26]. Furthermore, such banks can be implemented through a short-term Fourier transform (STFT), thereby allowing the integration of single microphone noise reduction algorithms based on a fast Fourier transform (FFT) [22,26]. However, the relevant literature pertaining to STFT-based single-channel noise reduction algorithms such as STSA, NNSC, and DNN for a uniform polyphase DFT filter bank is limited.

The remaining paper is structured as follows. Section 2 describes the uniform polyphase DFT filter bank used to implement the proposed wearable hearing device spectral gain enhancement method. Section 3 provides a review of a conventional spectral gain estimation method based on a Wiener filter with a DD-based approach. Section 4 describes the NNSC–Wiener filter for speech enhancement. Section 5 describes the efficiency validation of the proposed approach, through the perceptual evaluation of the speech quality (PESQ) [27] and comparison with the NNSC [18], a two-stage Mel-warped Wiener filter [9], and a model-based Wiener filter [10]. Section 6 presents the concluding remarks.

## 2. Hearing Device Spectral Enhancement

An auditory filter bank must have equally spaced narrow frequency bands and at least 60 dB of stopband attenuation (a higher value is ideal) [21], as mentioned in Section 1. Furthermore, a filter bank must exhibit low computational complexity and a small time delay of less than 10 ms. These constraints can be satisfied using a uniform polyphase DFT filter bank, implemented through the FFT. We introduce a filter bank of 32 channels with a time delay of 8 ms under a sampling rate of 16 kHz [21,28,29].

**Figure 1:**
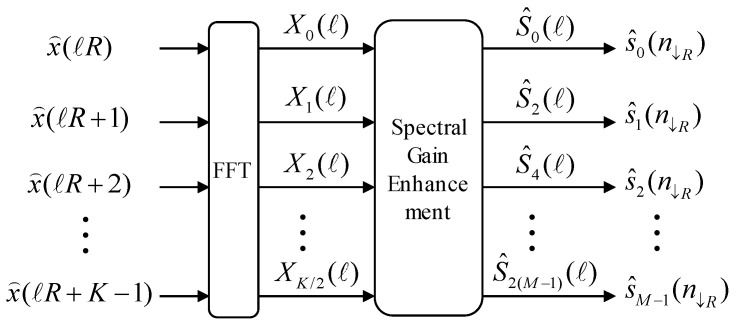
The block diagram of the analysis stage of a hearing device filter bank with a spectral enhancement algorithm.

**Figure 2:**
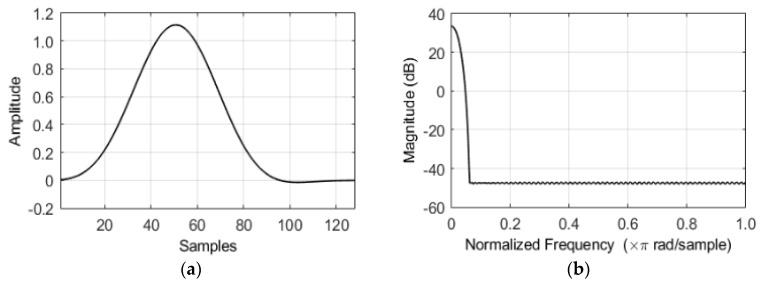
(**a**) The impulse response of a low pass filter prototype with 128 sample points and (**b**) its magnitude spectra at the normalized frequencies. The relative sidelobe attenuation is −80.5 dB.

As shown in Figure 1, the filter bank is implemented by setting the number of channels M=32, the downsample factor R=16, and the FFT size K=128 to satisfy the oversampled perfect reconstruction condition with a time delay of 8 ms. The ℓth input frame signal $x_{\ell }={\left[x\left(\ell R\right),\;x\left(\ell R+1\right),x\left(\ell R+2\right),\ldots ,x\left(\ell R+K-1\right)\right]}^{T}$ is generated by buffering the input time-discrete signal x(n), where T is the transpose operator. Furthermore, by implementing an FFT, the prototype low pass filter (LPF) applied signal on $x_{\ell }$ and ${\hat{x}}_{\ell }$ is converted into the complex-spectral value $X_{k}\left(\ell \right)$ in the kth frequency bin (k=0,1,…, K−1) and ℓth frame. The prototype LPF is developed using the method described in [3]. The definitions of the 128 sequences and form of the frequency domain magnitude are illustrated in the upper and lower panels in Figure 2, respectively. 

The enhanced version of the spectral value $X_{k}\left(\ell \right)$**,** ${\hat{S}}_{k}\left(\ell \right)$**,** is obtained by applying a spectral enhancement algorithm to $X_{k}\left(\ell \right)$ in the kth frequency bin (k=0,1,…,K/2). Subsequently, the 16 down-sampled speech denoised signals in the mth frequency band,$\;{\hat{s}}_{m}\left(n_{↓16}\right),$ can be extracted from the real number part of the complex value ${\hat{S}}_{k=2m}\left(\ell \right)$. These signals are then utilized to obtain the power envelope of each band. The term $Y_{k}\left(\ell \right)$ denotes the corresponding spectral output of the hearing aid algorithms, such as a dynamic range compressor and a feedback cancellation algorithm, to ${\hat{S}}_{k=2m}\left(\ell \right)$ and can be converted into the ℓth frame signal $y_{\ell }={\left[y\left(\ell R\right),\;y\left(\ell R+1\right),y\left(\ell R+2\right),\ldots ,y\left(\ell R+K-1\right)\right]}^{T}$ through an inverse FFT [20,21,22]. Finally, the filter-bank-synthesized output signal is derived from the overlap-and-add operation of the LPF applied signal at $y_{\ell }$. 

## 3. Conventional Spectral Gain Estimation

This section provides the review of a conventional spectral gain estimation method based on the STFT. When the target speech s(n) is deteriorated by additive noise d(n), the noisy speech x(n) is related to s(n) and d(n) in the frequency domain as $X_{k}\left(\ell \right)=S_{k}\left(\ell \right)+D_{k}\left(\ell \right),$ where $X_{k}\left(\ell \right),$ $S_{k}\left(\ell \right),$ and $D_{k}\left(\ell \right)$ are the spectral components of x(n), s(n), and d(n), respectively, at the kth frequency (k=0,1,…, K−1) and ℓth frame (ℓ=0,1,2,…). 

**Figure 3:**
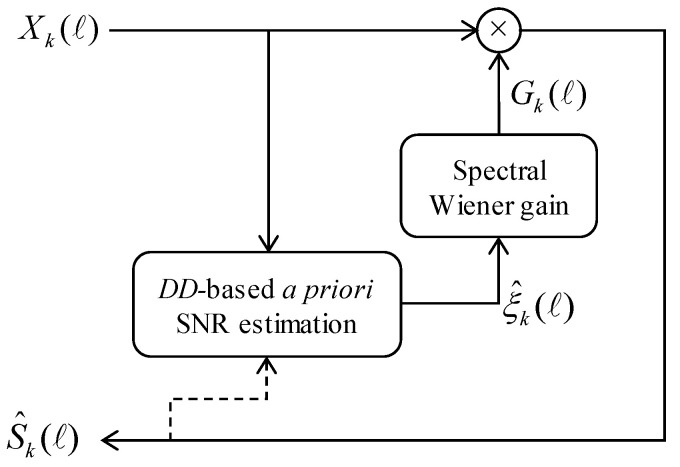
The block diagram of a conventional Wiener filter based on decision-directed (DD) a priori signal-to-noise ratio (SNR) estimation.

As shown in Figure 3, the spectral gain for speech enhancement, $G_{k}\left(\ell \right),$ attempts to estimate $S_{k}\left(\ell \right)$ in the form ${\hat{S}}_{k}\left(\ell \right)=G_{k}\left(\ell \right)X_{k}\left(\ell \right)$. Here, $G_{k}\left(\ell \right)$ can be represented in the form of the following Wiener filter [8]:

(1) $$G_{k}\left(\ell \right)=\frac{{\hat{\xi }}_{k}\left(\ell \right)}{{\hat{\xi }}_{k}\left(\ell \right)+1}$$

where ${\hat{\xi }}_{k}\left(\ell \right)$ is the a priori SNR estimate and ${\hat{\xi }}_{k}\left(\ell \right)$ is processed according to the DD approach [8,9,10]:

(2) $${\hat{\xi }}_{k}\left(\ell \right)=max\left[\frac{{\hat{\xi }}_{k}^{DD}\left(\ell \right)}{{\hat{\xi }}_{k}^{DD}\left(\ell \right)+1}\cdot \frac{\left|X_{k}\left(\ell \right)\right|}{\sqrt{{\hat{\lambda }}_{D,k}\left(\ell \right)}},TH_{\xi }\right]$$

where $TH_{\xi }$ and ${\hat{\lambda }}_{D,k}\left(\ell \right)$ denote the prefixed minimal threshold value and noise variance estimate, respectively. Moreover,

(3) $${\hat{\xi }}_{k}^{DD}\left(\ell \right)=\;\beta_{\xi }\cdot \frac{{\hat{S}}_{k}\left(\ell -1\right)}{\sqrt{{\hat{\lambda }}_{D,k}\left(\ell -1\right)}}+\left(1-\beta_{\xi }\right)\cdot \max\left[\frac{\left|X_{k}\left(\ell \right)\right|}{\sqrt{{\hat{\lambda }}_{D,k}\left(\ell \right)}}-1,\;0\right]\;$$

where $\beta_{\xi }\left(0\leq \beta_{\xi }<1\right)$ is a smoothing parameter, used to avoid the sudden adjustment of the SNR.

Due to its simplicity and effectiveness, ${\hat{\xi }}_{k}\left(\ell \right)$ in (2) is commonly used to suppress the noise components; however, because this value is directly obtained from the noisy speech $X_{k}\left(\ell \right),$ it may be inaccurate in severely noisy environments [2]. Moreover, because the accuracy of ${\hat{\xi }}_{k}^{DD}\left(\ell \right)$ is affected by the previous target speech estimate $\left|{\hat{S}}_{k}\left(\ell -1\right)\right|,$. the error in estimating ${\hat{\xi }}_{k}^{DD}\left(\ell \right)$ may propagate to the estimation error of the spectral gain $G_{k}^{DD}\left(\ell \right)$, resulting in the distortion of the estimated target speech. To address this problem, we integrate the DD method with the NNSC strategy to develop a novel spectral gain enhancement stage.

## 4. Proposed Spectral Gain Enhancement Driven by NNSC-Based Residual Noise Reduction

The proposed method is aimed at minimizing the residual noise remaining after DD-based Wiener filter processing. In particular, the NNSC approach, which minimizes the Gaussian independent identically distributed noise, is used to remove the whitened residual noise. Subsequently, the NNSC reconstructs the target speech spectra of the Wiener filter output by using a pre-trained dictionary and removing the whitened residual noise components. In other words, the proposed method enhances the DD-based spectral gain $G_{k}\left(\ell \right)$ in (1) through an NNSC technique to address the residual noise components that remain after applying $G_{k}\left(\ell \right)$. 

**Figure 4:**
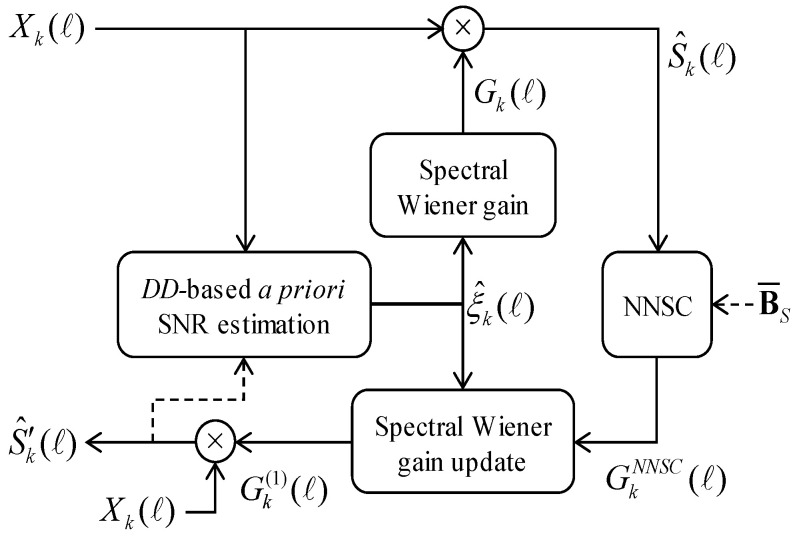
The block diagram of the proposed spectral gain enhancement driven by non-negative sparse coding (NNSC)-based residual noise reduction.

As shown in Figure 4, in the first stage, the DD-based a priori SNR and spectral enhancement gain are estimated based on the Wiener filter described in Equation (1). In the second stage, the NNSC method strategy is applied to increase the spectral gain $G_{k}\left(\ell \right)$ estimated in the first stage. 

To increase $G_{k}\left(\ell \right)$ in (1) through the NNSC technique, $\left|{\hat{S}}_{k}\left(\ell \right)\right|\left(=G_{k}\left(\ell \right)\left|X_{k}\left(\ell \right)\right|\right)$. is first accumulated over all the frequency bins as a vector ${\hat{S}}_{\langle K+1\rangle }$. ${\hat{S}}_{\langle K+1\rangle }={\left[\left|{\hat{S}}_{0}\left(\ell \right)\right|,\left|{\hat{S}}_{1}\left(\ell \right)\right|,\ldots ,\left|{\hat{S}}_{K-1}\left(\ell \right)\right|\right]}^{T}$, where T is the transpose operator. Subsequently, ${\hat{S}}_{\langle K+1\rangle }$ can be expressed in terms of the pre-trained basis matrix ${\bar{B}}_{S\langle K\times N\rangle }$ and an activation vector $a_{S\langle K\times 1\rangle }$ as

(4) $${\hat{S}}_{\langle K+1\rangle }={\bar{B}}_{S\langle K\times N\rangle }a_{S\langle K\times 1\rangle }+e_{\langle K\times 1\rangle }$$

where $e={\left[e_{0},e_{1},\ldots ,e_{K-1}\right]}^{T}$ is the vector consisting of the residual noise components remaining after applying over all frequency bins, and the subscripts 〈·〉 represent the matrix (or vector) dimension. ${\bar{B}}_{S}$ is trained from a universal speech DB by assuming that ${\bar{B}}_{S}$ can reconstruct any clean target speech. The main task in the NNSC framework is to determine $a_{S}$ that can minimize the error $e_{S}\left(=\hat{S}-{\bar{B}}_{S}a_{S}\right)$ by minimizing the cost function with the L1 sparsity constraint [18,19]:

(5) $${\bar{a}}_{S}=\underset{a_{S}}{\arg\;\min}D\left(\hat{S}|{\bar{B}}_{S}a_{S}\right)+\lambda {\left|a_{S}\right|}_{1}$$

where $D\left(\hat{S}|{\bar{B}}_{S}a_{S}\right)$ is either the Euclidean (EU) distance or the Kullback–Leibler (KL) divergence, and λ is a sparseness control parameter. The term $a_{S}$ in Equation (5) is estimated via random initialization and iterative updating using an iteration number (*iter*), such that the error e is minimized and converges:

(6) $$a_{S}^{\left(iter+1\right)}=a_{S}^{\left(iter\right)}⊗\frac{{\bar{B}}_{S}^{T}\hat{S}}{{\bar{B}}_{S}^{T}{\bar{B}}_{S}a_{S}^{\left(iter\right)}+\lambda }$$

where the multiplication, ⊗, and division operators are element-wise operators. Finally, we obtain the NNSC-based spectral gain to attenuate the residual noise remaining after applying the Wiener filter as

(7) $$G_{k}^{NNSC}\left(\ell \right)=\frac{{\hat{\eta }}_{k}\left(\ell \right)}{{\hat{\eta }}_{k}\left(\ell \right)+1}$$

where

(8) $${\hat{\eta }}_{k}\left(\ell \right)=\frac{\sum_{r=1}^{N}{\bar{B}}_{S}^{\left[k,r\right]}a_{S}}{\max\left[e_{k},\varepsilon \right]}$$

and where  ε is the minimum value that can avoid a zero value in the numerator. 

Next, the $G_{k}^{NNSC}\left(\ell \right)$ obtained using Equation (7) is used to improve $G_{k}\left(\ell \right)$ by using two approaches. In the first approach, $G_{k}\left(\ell \right)$ is redefined by applying $G_{k}^{NNSC}\left(\ell \right)$ as a weight to the a priori SNR estimate ${\hat{\xi }}_{k}\left(\ell \right)$, as

(9) $$G_{k}^{\left(1\right)}\left(\ell \right)=\frac{{\hat{\xi }}_{k}\left(\ell \right)G_{k}^{NNSC}\left(\ell \right)}{{\hat{\xi }}_{k}\left(\ell \right)G_{k}^{NNSC}\left(\ell \right)+1}$$

In the second approach, $G_{k}^{NNSC}\left(\ell \right)$ and $G_{k}\left(\ell \right)$ in Equation (1) are multiplicatively combined as

(10) $$G_{k}^{\left(2\right)}\left(\ell \right)=G_{k}\left(\ell \right)G_{k}^{NNSC}\left(\ell \right)$$

According to the results of a preliminary speech enhancement experiment performed considering Equations (9) and (10), the quality of enhanced speech through $G_{k}^{\left(1\right)}\left(\ell \right)$ is comparable to or slightly higher than that by $G_{k}^{\left(2\right)}\left(\ell \right)$ for all the SNRs. Thus, the performance evaluation is conducted using $G_{k}^{\left(1\right)}\left(\ell \right)$, as described in Section 5. 

Finally, the enhanced speech spectral estimate is calculated as ${\hat{S}}_{k}^{\prime }\left(\ell \right)=G_{k}^{\left(1\right)}\left(\ell \right)X_{k}\left(\ell \right)$, which is later used to estimate ${\hat{\xi }}_{k}$ in Equations (2) and (3) recursively. 

## 5. Performance Evaluation

The performance of the proposed hearing device spectral gain enhancement algorithm based on NNSC and the Wiener filter was evaluated by measuring the PESQ scores [27]. The test set involved 240 speech utterances from the TIMIT DB [30] and four types of noise sources (Gaussian, babble, factory, and car) from the NOISEX-92 DB [31]. The noise signals were mixed with the target speech at different SNRs ranging from −5 to 20 dB in steps of 5 dB. Each signal was sampled at 16 kHz and was segmented using a 128-point LPF, as shown in Figure 2; each segment had an overlap of one-eighth with the previous segment.

To implement the NNSC technique, 100 speech basis vectors were trained using the speech utterances in the TIMIT DB, with the training data including the speech of sixteen male and eight female speakers, with a duration of approximately 560 s. The number of speech basis vectors (100) was selected with reference to [16]. The speakers in the basis training set did not overlap with those in the test set. The noise variance ${\hat{\lambda }}_{D,k}\left(\ell \right)$ in Equations (2) and (3) was updated as ${\hat{\lambda }}_{D,k}\left(\ell \right)=0.95\;{\hat{\lambda }}_{D,k}\left(\ell \right)+0.05\;{\left|X_{k}\left(\ell \right)\right|}^{2}$ in the noise-only intervals, as in [8].

**Figure 5:**
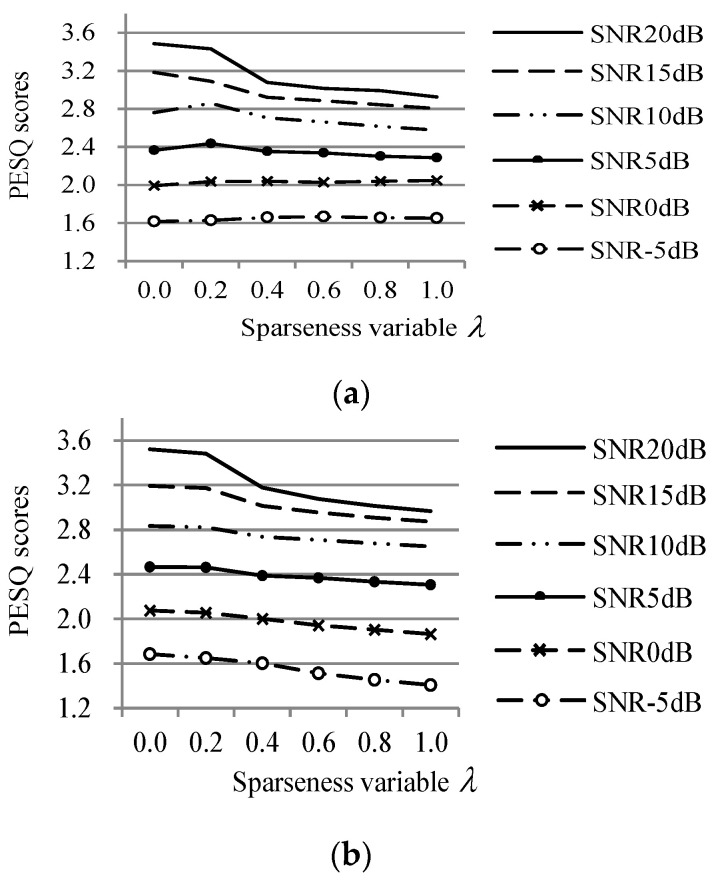
The comparison of perceptual evaluation of the speech quality (PESQ) scores for the proposed method for different sparseness control parameters, under (**a**) babble and (**b**) Gaussian noise conditions.

First, to determine the optimal value of the sparseness parameter λ in Equations (5) and (6) to optimize the speech quality performance, the PESQ scores of the speech signals produced using the proposed method were measured by changing λ from 0 to 1, as shown in Figure 5. The evaluation was performed using the training data, and the proposed method was implemented using the EU distance. According to the results, the proposed method achieved the highest PESQ scores averaged over all the SNRs when λ was set to 0.2. Therefore, λ was set as 0.2 in the subsequent experiments.

Second, the effect of the two different cost functions in Equation (5) on the speech enhancement performance was investigated. Table 1 presents a comparison of the PESQ scores of the conventional Wiener filter and the proposed method, with scores presented for cases involving the EU distance and KL divergence in the proposed method. Both the KL-NNSC and EU-NNSC achieved higher PESQ scores than that of the Wiener filter for all the SNRs. At high SNRs (20 dB), the PESQ scores of the EU-NNSC and KL-NNSC were comparable. However, the EU-NNSC scores were significantly higher than those of the KL-NNSC for lower SNRs (from −5 to 15 dB). This finding indicates that the EU-NNSC is likely a more appropriate form for the proposed NNSC-Wiener filter method than the KL-NNSC form. In particular, the residual noise remaining after the application of the Wiener filter may be Gaussian-distributed [9,10], and the EU-NNSC aimed to find the basis and activation components by extracting the Gaussian independent identically distributed noise [18,19].

Third, as described previously, the proposed method was implemented in an online auditory device filter bank framework. Thus, it was necessary to examine the difference in the performance of the online and offline implementations of the proposed method. In the offline implementation, the NNSC strategy, as described in Section 4, was applied to each utterance instead of to each frame. The PESQ scores of the offline implementation were evaluated under the babble and Gaussian noise conditions.

Table 2 presents a comparison of the PESQ scores for the online and offline implementations. The PESQ scores for the offline implementation were slightly higher than those for the online implementation under both the noise conditions, because the offline implementation could obtain more accurate estimates for the activation vectors than the estimates obtained for the online implementation. Nevertheless, the performance difference between the online and offline implementations was minimal, indicating that the block size of the accumulated signal for the NNSC did not significantly affect the speech enhancement performance in the proposed method.

**Figure 6:**
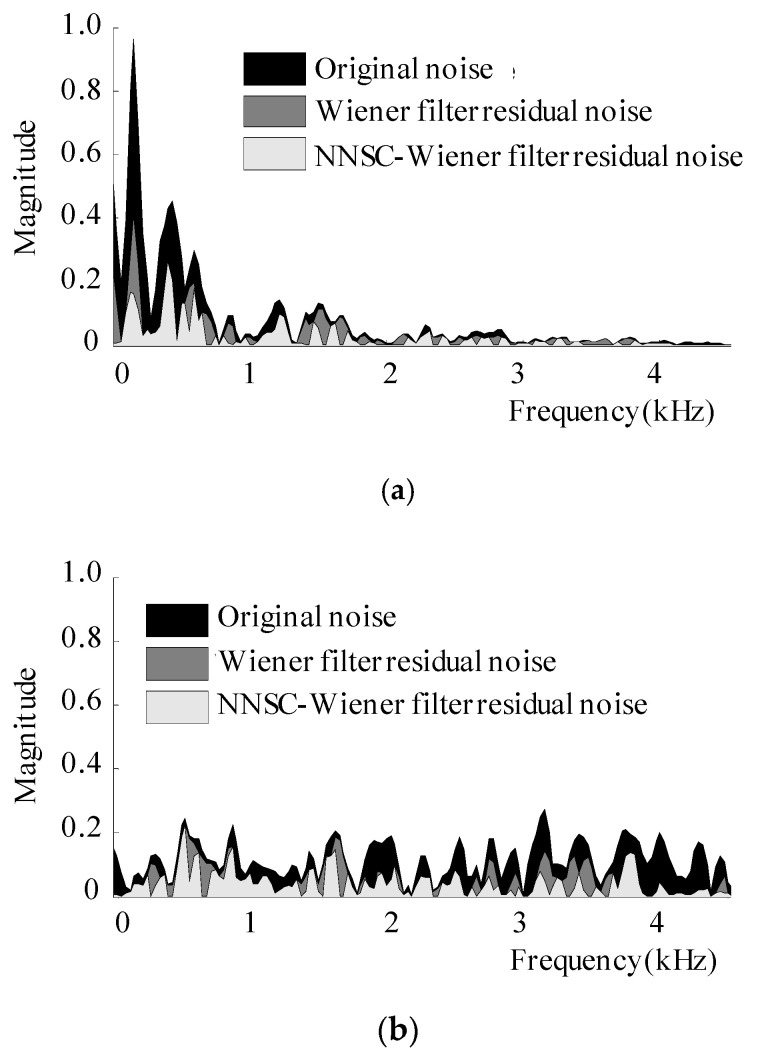
The comparison of the spectral residual noise remaining after the application of the conventional Wiener filter and proposed NMF-based Wiener filter method to (**a**) babble and (**b**) Gaussian noisy speech at an SNR of 5 dB.

Fourth, the effectiveness of the proposed method in reducing the residual noise remaining after the application of the Wiener filter is demonstrated in Figure 6. Each black area represents the spectral magnitude of the babble or Gaussian noise of one frame, which was mixed to clean the speech at an SNR of 5 dB. The noise reduction attained by the Wiener filter and proposed method is indicated by the dark gray and light gray areas, respectively. The proposed method achieved a lower spectral magnitude noise than that when only the Wiener filter was used.

**Figure 7:**
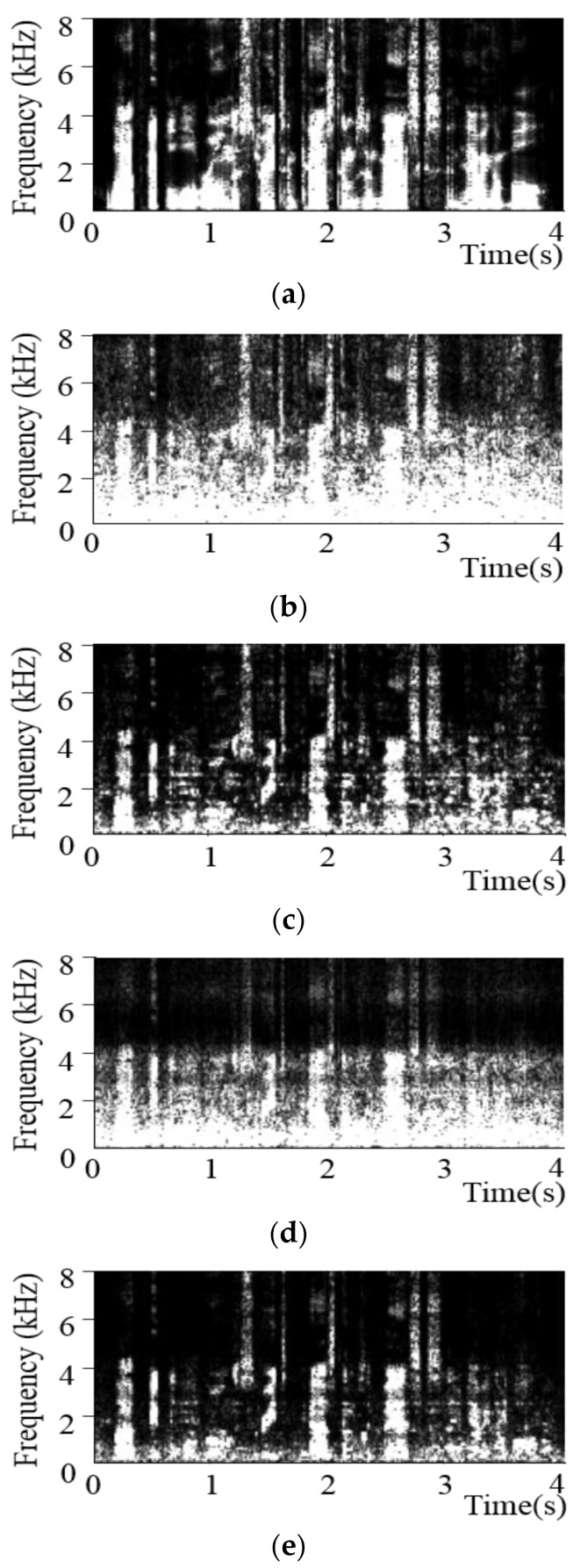
Spectrograms for a sample sentence pertaining to (**a**) clean speech, (**b**) speech in babble noise at an SNR of 0 dB, and those for the speech signals processed using (**c**) $G_{k}\left(\ell \right)$ (**d**) NNSC processing with results based on [17], and (**e**) the proposed method based on $G_{k}^{\left(1\right)}\left(\ell \right)$.

Fifth, the spectrograms of the speech signal enhanced using the proposed and existing methods were compared, as shown in Figure 7. Figure 7a,b illustrates the spectrograms of the desired clean speech and its noise-contaminated version, respectively, at an SNR of 0 dB under the babble noise condition. Figure 7c,d shows the results obtained by applying the Wiener filter in (1) and NNSC to the signal in Figure 7b, respectively. Figure 7e shows the results obtained using the proposed NNSC–Wiener filter. The proposed method effectively suppressed the babble noise components. As shown in Figure 7c, some residual noise remained after processing through a Wiener filter. However, the proposed method could successfully suppress most of the noise spectra, as shown in Figure 7e.

Table 3 compares the PESQ scores [26] corresponding to the spectrograms presented in Figure 7. Although it was expected that the speech enhancement performance when using the Wiener filter (Figure 7c) would be superior to that of the NNSC (Figure 7d), the PESQ scores for the two approaches were comparable. This aspect is likely why the Wiener filter led to target speech distortion in addition to the residual noise problem. The PESQ scores for the proposed method, in which both the NNSC strategy and Wiener filter were applied, were considerably higher than those for the Wiener filter or NNSC-based speech enhancement method. This finding demonstrated that the combination of the NNSC and Wiener filter could enhance the performance by addressing the target speech distortion and residual noise problems.

Finally, the speech enhancement performance of the proposed approach was assessed and compared with those of the original noisy speech (**No**), the NNSC technique (**R1**) [18], the two-stage Mel-warped Wiener filter (**R2**) [9], and the model-based Wiener filter (**R3**) [10]. In this experiment, four distinct types of noise were considered, and a statistical analysis was conducted using a Games–Howell criterion-based multiple-pair test. Table 4 presents the comparison of the PESQ scores averaged over all the SNRs ranging from −5 to 10 dB. **R2** exhibited the highest performance in Gaussian noise environments, and **R3** exhibited the highest performance under the babble, factory, and car noise conditions. However, the proposed method outperformed all the other approaches under all the noise conditions, except under the car noise condition, in which case, the performance of the proposed method was statistically comparable to that of **R3**. 

The number of iterations in Equation (6) to estimate $a_{S}$ is a crucial parameter in terms of the computational complexity for real-world applications. In our experiments, the number of iterations was approximately 13, averaged over all frames (minimum 3, maximum 39). 

## 6. Conclusions

The proposed method was aimed at enhancing the conventional spectral Wiener filter approach in terms of the hearing device speech enhancement performance by introducing the NNSC approach to reduce the residual noise after the application of the Wiener filter. To this end, the NNSC technique was combined with the a priori SNR estimate to enhance the gain attenuator of the Wiener filter. The spectral gain was increased using the proposed approach through a uniform polyphase DFT filter bank to fulfill the low computational complexity and algorithm processing delay criteria for hearing devices.

Subsequently, the performance of the proposed speech enhancement method was compared to those of the conventional Wiener filter, two-stage Mel-warped Wiener filter, and conventional NNSC method in terms of the PESQ scores and spectrograms. The results indicated that the proposed method produced significantly higher PESQ scores than the other methods for all the SNRs under four different noise conditions.

Accordingly, it could be concluded that the proposed algorithm did not notably increase the computational costs. To further decrease the computational costs according to the target hearing device system, the NNSC can be applied to function selectively according to the noise environments. Nevertheless, the development of the NNSC controller is beyond the scope of this work and should be discussed in future studies.

The current research on speech processing has been focused on DNN techniques. Notably, the DNN-based speech enhancement method can outperform the existing methods. Nevertheless, this approach involves substantially higher computing costs. Thus, it is difficult to implement DNN-based approaches in portable hearing devices that require a low computing complexity for real-world implementations. In this regard, it may be desirable to combine the existing Wiener filter and DNN approaches to enhance the performance in terms of both the speech quality and intelligibility.

Effective hearing wearable devices are expected to be of notable objective value as a natural connection to other devices. Specifically, from the viewpoint of sustainable internet-of-things wearables, hearing wearable devices represent an essential element in recognizing user contexts to construct human-oriented environments [32,33,34]. This study was aimed at improving the speech quality enhancement performance of the existing Wiener filter, implemented through hearing wearable device filter bank algorithms. However, the relevant literature on the NNSC or DNN-based speech enhancement algorithms for the filter bank is limited. The presented findings may provide guidance to achieve a satisfactory speech processing performance for successful hearing wearable devices.

## Figures and Tables

**Table 1:** The comparison of PESQ scores for the conventional Wiener filter and two different NNSC cost functions under a babble noise condition.

SNR (dB)	Wiener	Wiener + KL-NMF	Wiener + EU-NMF
20	3.345	3.363	3.358
15	2.996	3.02	3.033
10	2.628	2.659	2.693
5	2.247	2.281	2.344
0	1.854	1.891	1.987
−5	1.434	1.467	1.621

The 95% confidence intervals range from 0.019–0.026.

**Table 2:** The comparison of PESQ scores for the online and offline implementations of the proposed method under babble and Gaussian noise conditions.

SNR (dB)	Babble		Gaussian	
	Online	Offline	Online	Offline
20	3.360	3.384	3.492	3.418
15	3.035	3.055	3.122	3.091
10	2.695	2.716	2.759	2.778
5	2.346	2.374	2.391	2.449
0	1.991	2.021	2.024	2.104
−5	1.623	1.644	1.658	1.744

**Table 3:** The PESQ scores pertaining to the spectrograms shown in Figure 7.

Method	PESQ
Noisy speech (Figure 7b)	1.771
Wiener filter (Figure 7c)	1.892
NNSC (Figure 7d)	1.891
Proposed NNSC–Wiener filter (Figure 7e)	**2.021**

**Table 4:** The comparison of PESQ scores for different approaches, averaged over [10, 5, 0, −5] SNRs under various noise conditions: **No**: no processing, **R1**: NNSC [18], **R2**: two-stage Mel-warped Wiener filter [9], **R3**: model-based Wiener filter [10], and **PR**: proposed method.

Method	Noise Type			
	Babble	Gaussian	Factory	Car
**No**	1.869 (**S**)	1.695 (**S**)	1.740 (**S**)	3.300 (**S**)
**R1**	1.750 (**S**)	0.355 (**S**)	1.672 (**S**)	2.643 (**S**)
**R2**	1.916 (**S**)	2.143 (**NS**)	1.816 (**S**)	3.485 (**S**)
**R3**	2.029 (**S**)	2.060 (**S**)	1.956 (**S**)	3.730 (**NS**)
**PR**	2.162	2.203	2.133	3.677

‘**S**’ and ‘**NS**’ in the parentheses indicate the difference between the proposed method and each counterpart, pertaining to the Games–Howell test (**S**: significant, **NS**: not significant).

## Abbreviations

List of abbreviations and corresponding definitions.

DB	database
DD	decision-directed
DFT	discrete Fourier transform
DNN	deep neural network
EU	Euclidean
FFT	fast Fourier transform
HI	hearing-impaired
KL	Kullback–Leibler
LPF	low pass filter
NMF	non-negative matrix factorization
MMSE	minimum mean square error
NN	neural network
NNSC	non-negative sparse coding
PESQ	perceptual evaluation of speech quality
PSD	power spectral density
SNR	signal-to-noise ratio
STFT	short-term Fourier transform
STSA	short-term spectral amplitude
